# Diacylglycerol Activates the *Drosophila* Light Sensitive Channel TRPL Expressed in HEK Cells

**DOI:** 10.3390/ijms24076289

**Published:** 2023-03-27

**Authors:** Elisheva Rhodes-Mordov, Tal Brandwine-Shemmer, Rachel Zaguri, Rita Gutorov, Maximilian Peters, Baruch Minke

**Affiliations:** Department of Medical Neurobiology, Institute for Medical Research Israel-Canada (IMRIC), Edmond and Lily Safra Center for Brain Sciences (ELSC), Faculty of Medicine, The Hebrew University, P.O. Box 12272, Jerusalem 91120, Israel

**Keywords:** *Drosophila* TRP/TRPL channels, Diacylglycerol (DAG), 1-oleoyl-2-acetyl-sn-glycerol (OAG), 3-hydroxypropane-1,2-diylbis(4-(4-((E)-(4-butylphenyl) diazenyl) phenyl, (OptoDArG), phospholipase C (PLC)

## Abstract

Physiological activation by light of the *Drosophila* TRP and TRP-like (TRPL) channels requires the activation of phospholipase Cβ (PLC). The hydrolysis of phosphatidylinositol 4,5, bisphosphate (PIP_2_) by PLC is a crucial step in the still-unclear light activation, while the generation of Diacylglycerol (DAG) by PLC seems to be involved. In this study, we re-examined the ability of a DAG analogue 1-oleoyl-2-acetyl-sn-glycerol (OAG) to activate the TRPL channels expressed in HEK cells. Unlike previous studies, we added OAG into the cytosol via a patch-clamp pipette and observed robust activation of the expressed TRPL channels. However, TRPL channel activation was much slower than the physiologically activated TRPL by light. Therefore, we used a picosecond-fast optically activated DAG analogue, OptoDArG. Inactive OptoDArG was added into the intracellular solution with the patch-clamp pipette, and it slowly accumulated on the surface membrane of the recorded HEK cell in the dark. A fast application of intense UV light to the recorded cell resulted in a robust and relatively fast TRPL-dependent current that was greatly accelerated by the constitutively active TRPL^F557I^ pore-region mutation. However, this current of the mutant channel was still considerably slower than the native light-induced TRPL current, suggesting that DAG alone is not sufficient for TRPL channel activation under physiological conditions.

## 1. Introduction

In *Drosophila* phototransduction upon photon absorption, rhodopsin is converted into the photopigment-active state, metarhodopsin. This leads to the activation of the heterotrimeric G protein by promoting a GDP to GTP exchange on the G_q_α subunit. In turn, this leads to the activation of phospholipase Cβ(PLC), which hydrolyzes a minor surface membrane phospholipid, phosphatidylinositol 4,5 bisphosphate (PIP_2_), into the water-soluble inositol 1, 4, 5-trisphosphate (IP_3_), the hydrophobic membrane bound diacylglycerol (DAG) and a proton. Subsequently, two classes of light-activated channels, transient receptor potential (TRP) that is highly permeable to Ca^2+^ and TRP-like (TRPL) that is a non-selective cation channel, open by a still-unclear mechanism [1]. PLC also promotes hydrolysis of the GTP bound to G_q_α (GTPase reaction), resulting in G_q_α bound to GDP, and this ensures the termination of G_q_α activity [2]. For reviews on the phototransduction cascade, see [3,4,5,6,7].

The retinal degeneration A (*rdgA*) gene that encodes for DAG kinase, the enzyme that inactivates DAG by phosphorylation [8], has turned out to be important for understanding TRP/TRPL channel activation. The *rdgA* mutant exhibits light-independent retinal degeneration [9] resulting from the constitutive activity of the light-activated channels [10]. These findings gave rise to the DAG hypothesis of TRP/TRPL channels activation, by which the DAG generated by the phototransduction cascade acts as the second messenger for channel activation. Importantly, the partial rescue of the degeneration was achieved by eliminating the TRP channel in the double mutant, having both null mutation in *trp* and strong mutation in *rdgA* (*rdgA*^1^;;*trp^P^*^343^, [10]). Moreover, it was shown that the double mutant, having a virtually null mutation in PLC and a strong mutation in *rdgA* (*norpA^P^*^24^,*rdgA*^1^;;), partially rescued the light response in the virtually null *norpA^P^*^24^ mutant, further supporting the hypothesis that DAG is involved in channel activation [11]. However, the application of DAG analogues to the bathing solution of isolated ommatidia during whole-cell patch clamp recordings did not activate the channels (Hardie personal communication).

Additional studies on TRP/TRPL channels’ activation by DAG were carried out in tissue culture cells. However, there was a marked difference in the outcome of the heterologous expression between the TRP and TRPL channels. While there is wide agreement that the expression of the TRPL channels resulted in functional TRPL channels in the host cells [12], this is not the case for the TRP channel. The few reports on TRP expression seem to indicate that the expressed channels are not functionally active [13,14,15]. Therefore, further studies on in vitro expressed channels were carried out only on TRPL channels expressed in tissue culture cells in several expression systems [16,17,18,19,20,21,22]. The main functional characteristics and activation of the expressed TRPL channels are, in general, similar to those of the native *Drosophila* TRPL channels [17,21].

In spite of the failure to activate isolated *Drosophila* ommatidia by exogenous application of DAG analogues to the bathing solution (Hardie personal communication and our own unpublished data), the application of OAG at a low concentration (2 µM) to inside-out patches excised from the signaling compartment (rhabdomere) of dissociated *Drosophila* ommatidia resulted in significant but slow channel activation [23,24]. In contrast, the exogenous bath application of the DAG analogues OAG or 1-stearoyl-2-arachidonyl-*sn*-glycerol (SAG) to TRPL channels expressed in tissue culture cells did not activate the channels, while the activation of the mammalian TRPC3 by DAG analogues served as a positive control [20]. Since attempts to activate TRP and TRPL channels with exogenous DAG have been unsuccessful, the DAG hypothesis of TRP/TRPL channel activation has been largely abandoned [25].

In the present study, we re-examined the ability of a DAG analogue to activate the expressed TRPL channels in HEK cells. Unlike previous attempts to activate both the native and expressed TRPL channels by OAG application to the bathing solution, in this study we applied the DAG analogue to the cytosol via a patch clamp pipette. Strikingly, intracellular application of the DAG analogue robustly activated the expressed TRPL channels. However, TRPL channel activation was much slower than the physiologically activated TRPL channels by light. Therefore, we used a picosecond-fast [26] optically activated DAG analogue, OptoDArG [27]. Inactive OptoDArG slowly accumulated in the surface membrane in the dark. The fast application of intense UV light to the recorded cell resulted in a robust and relatively fast TRPL-dependent current that was greatly accelerated by a constitutively active TRPL^F557I^ pore-region mutation. However, even the measured OptoDArG-activated mutant-current was still considerably slower than the native light-induced TRPL current, suggesting that DAG alone is not sufficient for TRPL channel activation under physiological conditions.

## 2. Results

### 2.1. Heterologous Expression of the TRPL Channel in HEK Cells Resulted in Localization on the Surface Membrane and in Robust Functional Currents via an Endogenous Cascade

**Figure 1:**
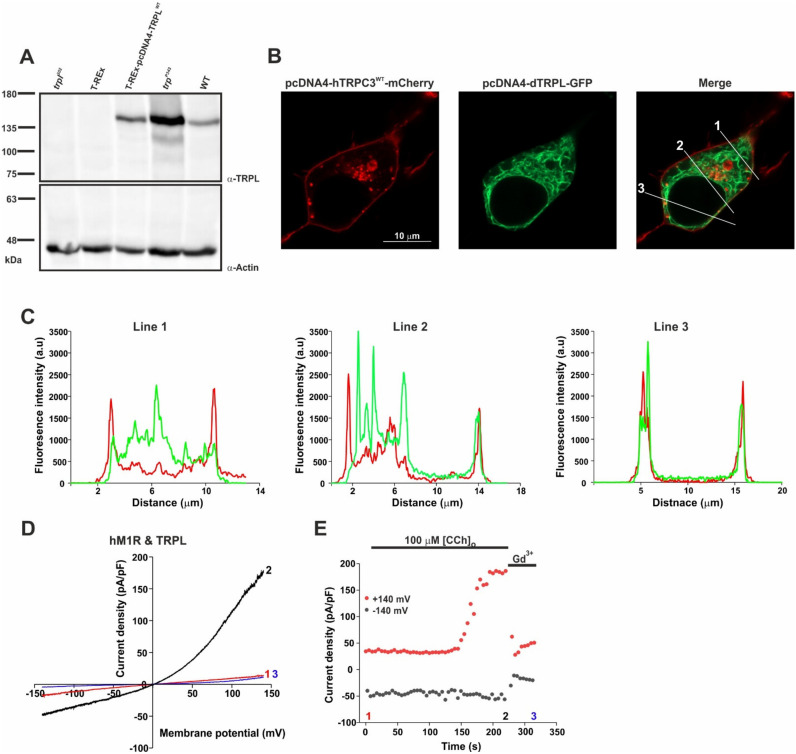
Heterologous expression, localization, and activation of the TRPL channel in HEK cells. (**A**) Western-blot analysis of T-REx-293 cells expressing dTRPL^WT^ (third lane), using α-dTRPL antibody. The *Drosophila trpl*^302^ mutant fly heads lacking the TRPL channel (first lane) and un-transfected T-REx cells (second lane) were used as negative controls. The *Drosophila trp^P^*^343^ mutant fly heads expressing only the TRPL channel (fourth lane), and the *Drosophila* WT fly heads (fifth lane) were used as positive controls. α-Actin antibody was used as protein loading control (*n* = 4). Molecular mass marker (in kDa) is indicated to the left of the gel. (**B**) Representative confocal fluorescence images of Naïve T-REx-293 cells co-transfected with hTRPC3^WT^-mCherry (red, left) and with dTRPL-GFP (green, middle). The right image shows the merged localization of hTRPC3^WT^-mCherry together with dTRPL^WT^-GFP. Scale bar 10 μm (applicable for all three images). The three lines crossing the cell (1, 2, 3) indicate the location of the analysis performed in section C. The total number of analyzed cells is 12. (**C**) Line profile graphs of the fluorescent intensity along the three white lines crossing the plasma membrane in the right merged image (1, 2, 3). The red graph represents hTRPC3^WT^-mCherry expression, and the green graph represents dTRPL^WT^-GFP expression. (**D**) Representative current–voltage relationship (i-V curves) obtained from patch clamp whole cell current measurements from a T-REx-293 cell heterologously expressing dTRPL^WT^, GFP and the human Muscarinic Receptor 1 (hM1R) in response to voltage ramps from −150 mV to +150 mV (in 1 s), before bath application of carbachol (CCh, red, 1), after application of 100 μM CCh (black, 2) and after application of Standard External Solution (SES, E1) to which 10 mM Gd^3+^ (TRPL channels blocker) was added (blue, 3). The total number of analyzed cells is 6. (**E**) Corresponding currents measured at +140 mV and −140 mV. Numbers indicate the time of the selected i-V curves.

**Figure 2:**
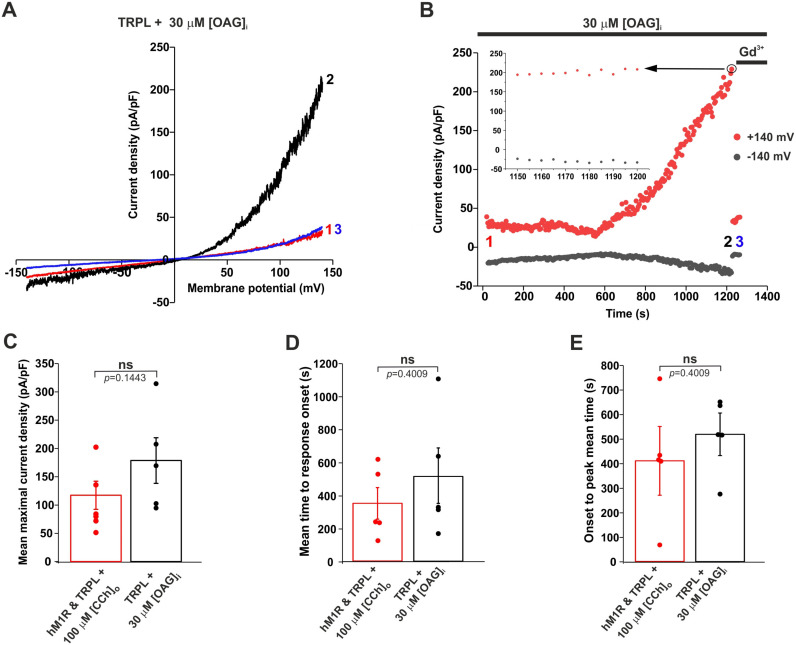
Activation of expressed TRPL channels by intracellular OAG. (**A**) Representative i-V curves obtained from patch clamp whole cell current measurements from a T-REx-293 cell expressing dTRPL^WT^ and GFP, in response to voltage ramps from −150 mV to +150 mV, during continuous intracellular application of 30 μM OAG, a short time (red, 1) and long time (black, 2) after whole cell formation and after application of E1, SES with 10 mM Gd^3+^ at the end of the experiment (blue, 3). (**B**) Corresponding currents at +140 mV and −140 mV. Numbers indicate the time of the selected i-V curves. The top red dot circled in black represents 13 superimposed dots, each at a time point of 5 s after the previous dot. *Inset*, an enlarged segment of the corresponding currents, from 1150 s to 1200 s, indicating no significant change in current density. (**C**) A bar chart showing the mean maximal current density measured at +140 mV after the bath application of 100 μM CCh in T-REx-293 cells expressing dTRPL^WT^, GFP and hM1R (red, *n* = 6, see Figure 1D,E). The bar chart also shows mean maximal current density measured at +140 mV after intracellular application of 30 μM OAG via the patch clamp pipette in cells expressing dTRPL^WT^ and GFP (black, *n* = 5, see Figure 3B). Error bars show SEM. *p* values were calculated using a two-tailed Mann–Whitney *U*-test and are indicated below the bars. Values from individual experiments are shown for each of the columns (circles). (**D**) A bar chart showing the mean time to response-onset measured after bath application of 100 μM CCh in T-REx-293 cells expressing dTRPL^WT^, GFP and hM1R (red, *n* = 6, see Figure 2B) or after whole cell formation with a pipette containing 30 μM OAG (intracellular application) in cells expressing dTRPL^WT^ and GFP (black, *n* = 5, see Figure 4B). Values from individual experiments are shown for each of the columns (circles). (**E**) A bar chart showing the time from the response onset to the maximal measured current (when no significant change in the i-V curve amplitude was observed during 50 s) in the same cell types as in the bar chart described in D. Error bars show SEM. *p* value was calculated using a two-tailed Mann–Whitney *U*-test and is indicated below the bar. Values from individual experiments are shown for each of the columns (circles).

The main functional characteristics of heterologously expressed TRPL channels are, in general, similar to those of the native *Drosophila* TRPL channels [17,21]. Nevertheless, because of the large variability in the reported magnitudes of the observed TRPL-dependent currents in different expression systems, we first established reference conditions that enabled the comparison of the current–voltage relationship (i-V curve) of expressed TRPL channels in HEK cells (see Materials and Methods). The heterologous expression of TRPL in HEK cells was readily obtained, as evidenced by Western blot analysis (Figure 1A) and by images of TRPL-GFP fluorescence. For the first time, TRPL-GFP fluorescence revealed the co-localization of plasma membrane expression of TRPL-GFP (Figure 1B,C, green, line 3) and hTRPC3-mCherry expression, which is known to express and delineate the plasma membrane of HEK cells ([28,29], Figure 1B,C, red, line 3). In all analyzed cells (*n* = 12), this co-localization was mostly present around the nucleus, whereas in other areas of the cell TRPL-GFP was mostly localized to the cytosol (Figure 1C, line 1 and line 2). To test for the functional expression of the fluorescently observed TRPL channel, we co-expressed the TRPL channels together with the human muscarinic M1 receptor (hM1R) and applied to the bath 100 μM carbachol (CCh) a potent agonist of the M1 receptor. This form of activation utilizes the major cascade proteins natively found in HEK cells, which can lead to TRPL activation (see scheme in Figure S4), but with a marked difference in stoichiometry and kinetics compared to photoreceptor cells. Upon the application of CCh, the expressed hM1R in HEK cells is known to activate endogenous heterotrimeric Gq protein, which in turn activates the endogenous PLCβ that activates the expressed TRPL channel [20]. When the expressed TRPL channels were activated by the application of CCh to the bath, the typical current–voltage relationships (i-V curves) were obtained by the application of voltage ramps (−150 to +150 mV applied during 1 s) during whole-cell patch clamp recordings from HEK cells co-expressing TRPL and hM1R (Figure 1D,E, CCh). The observed currents showed the typical outward rectification of the native TRPL channels, a reversal potential of ~0 mV, and the current could be blocked by the application of gadolinium (Gd^3+^, Figure 1D,E, for controls, see Supplement and Figure S1). The results further showed that it took a relatively long time (~5 min, Figure 1E and Figure 2D) for the CCh to activate the TRPL channels, as previously observed [20].

### 2.2. Application of a DAG Analogue to the Intracellular Solution of HEK Cells Robustly Activated the Expressed TRPL Channel

Due to our failures to activate expressed TRPL channels by the application of DAG analogues to the bathing solution ([20], Figure S2A,C), we examined our ability to activate the expressed TRPL channels by a DAG analogue applied to the cytosol. To this end, we performed whole cell voltage clamp recordings from HEK cells expressing TRPL channels and included 30 μM OAG in the intracellular pipette solution, so when the whole cell configuration was achieved, the OAG diffused into the cytosol. Strikingly, we observed a highly significant generation of outwardly rectifying typical TRPL-dependent i-V curves (Figure 2A–C, for controls, see Supplement and Figure S3), similar to the TRPL-dependent i-V curves induced by CCh application to cells co-expressing hM1R and TRPL (Figure 1D,E). However, the time of the initial activation of the TRPL dependent current by OAG (time to response onset, Figure 2D) and the time from the initial activation to the peak amplitude of the i-V curve (onset to peak time, Figure 2E) were relatively long. The peak amplitude is defined as the point when no significant change in the i-V curve amplitude was observed during 50 s of recording (as exemplified in Figure 2B, inset). The time to response onset and the time from the response onset to peak amplitude were not significantly different between the CCh and OAG activations (Figure 2D,E).

Although we showed the robust activation of expressed TRPL channels via a DAG analogue applied to the cytosol, this activation was much slower (~3 orders of magnitude) than the physiological activation of the TRPL/TRP channels by light in *Drosophila* (see below).

### 2.3. An Optically Activated DAG Analogue Accelerated the Kinetics of TRPL Channel Activation

A major difficulty in interpreting the observed activation of the TRPL channels by OAG in Figure 2 and by OAG at low concentration (2 µM) in inside-out patches excised from the signaling compartment (rhabdomere) of dissociated *Drosophila* ommatidia [23,24] is the very slow channel activation. We hypothesize that this slow activation reflects the accumulation time of a sufficient concentration of DAG necessary to activate the TRPL channels. Since the accumulation of exogenously applied OAG in the plasma membrane of both *Drosophila* rhabdomere and the surface membrane of HEK cells is inherently slow, we thought of overcoming this difficulty by using an optically activated DAG analogue.

The recent development of light-controlled lipid structures constituted a substantial technological advance that has enabled researchers to temporally control the active quantity of lipid mediators in membranes with a high degree of accuracy [27]. Groschner and colleagues synthetized OptoDArG, a photo-switchable DAG analogue containing two moieties of the arachidonic acid mimetic azobenzene side chain [27]. OptoDArG adopts the *trans* conformation in the dark and can be rapidly converted (in a picosecond timescale [26]) into the *cis* active conformation by UV light. In its *trans* form, OptoDArG (30 μM) was found to be functionally inert [27].

**Figure 3:**
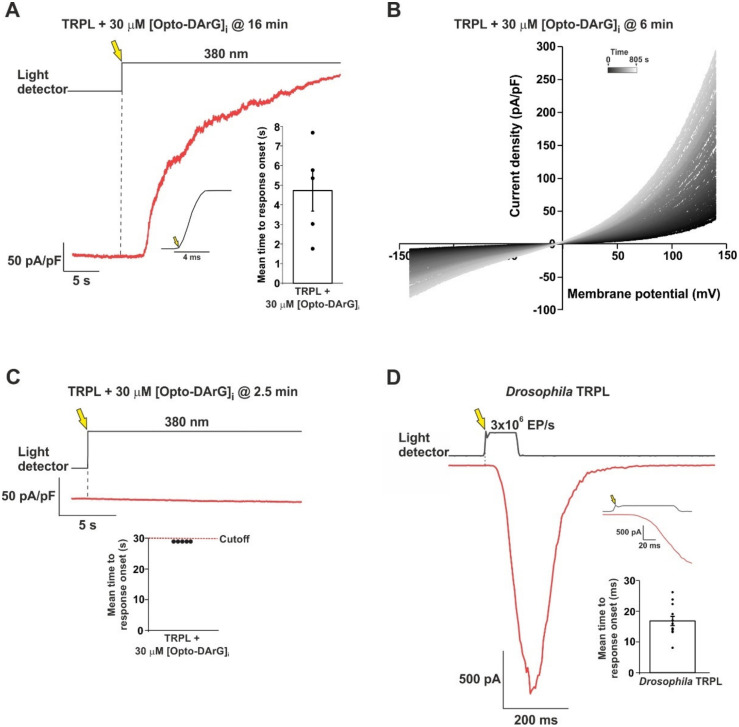
Activation of TRPL channels expressed in HEK cells by an optically controlled DAG analogue in comparison to light activation of *Drosophila* TRPL. (**A**) A representative current as a function of time showing an outward current induced by an intense UV (380 nm) light applied to an HEK cell expressing the TRPL channel, in which 30 μM OptoDArG was included in the intracellular solution of the patch clamp pipette. The UV light was applied 16 min after whole cell formation, while the cell was kept in the dark during that time to keep the OptoDArG in its inactive *trans* configuration. Then, the membrane voltage was increased in the dark from 0 mV to +100 mV holding potential and the UV light was turned on, resulting in a robust outward current (red trace). The upper black trace depicts the onset of the UV (380 nm) light measured by a light detector. The yellow arrow and the dashed line indicate the time of UV light onset. *Left inset,* the accurate open time of the electromechanical shutter, which allowed the accurate application of UV light as measured by the light detector*. Right inset*, a bar chart showing the mean time to response onset as measured from the turn-on time of the UV light to the initiation of the outward current (*n* = 5). Error bar shows SEM. Values from individual experiments are shown (circles). (**B**) A cluster of representative i-V curves obtained from patch clamp whole cell current measurements of a T-REx cell expressing TRPL, in response to voltage ramps from −150 mV to +150 mV, following intracellular application of 30 μM OptoDArG for 6 min (from whole cell formation) in the dark, followed by application of the intense UV light. The first trace (designated 0 s) is in black and indicates the UV 380 nm light onset. The time between each i-V curve is 5 s and the brightening of the trace colors indicates the progression of time during the recordings. The gradual increase in i-V curve amplitude presumably reflects the accumulation of active OptoDArG in the surface membrane. (**C**) A representative current as a function of time showing that negligible outward current was induced by the intense UV (380 nm) light applied to an HEK cell expressing the TRPL channel, in which 30 μM OptoDArG was included in the intracellular solution of the patch clamp pipette. The UV light was applied 2.5 min after whole cell formation, and was allowed 30 s to develop a response before recording was terminated. This time span was deemed the cutoff time (bottom inset). The upper black trace depicts the UV 380 nm light measured by the light detector. The yellow arrow and the dashed line indicate the time of UV light onset. *Inset*, a bar chart showing the upper limit of the cutoff time from UV light onset to the termination of the recording. During all 5 measurements, no response was evident within the cutoff time; therefore, there is no SEM. Values from individual experiments are shown (circles). (**D**) A representative trace showing whole cell current induced by orange light with intensity of 3 × 10^6^ EP/s (EF, effective photons) of the *Drosophila trp^P^*^343^ mutant photoreceptor (expressing only TRPL channels) in a patch clamp whole cell recording at a holding potential of −70 mV (red). The upper black trace depicts the light measured by the light detector. The yellow arrow and the dashed line indicate the time of orange light onset. *Top inset,* magnified waveform of the light stimulus as measured by the light detector and the initial light induced current*. Bottom inset*, a bar chart showing the mean time to response onset, from the time of light turn-on to the initial TRPL current (*n* = 12). Error bar shows SEM. Values from individual experiments are shown for each of the columns (circles).

To use OptoDArG in our experiments, we included 30 μM OptoDArG in the intracellular solution of the recording patch clamp pipette. After the formation of the whole cell configuration, we waited >10 min in the dark to allow for the diffusion of the inactive OptoDArG and its accumulation in the surface membrane of the recorded HEK cell. Then, we set the membrane voltage of the recorded cell to +100 mV, to enhance the TRPL-dependent current as a function of time. To confirm that the *trans* OptoDArG is functionally inert, we waited an additional ~30 s at +100 mV and observed no change in current, as previously found for the basal conductance in TRPC3-expressing cells [27]. After the fast opening of an electronic shutter (rise time ~4 ms, Figure 3A, inset) that illuminated the recorded cell with an intense UV (380 nm) light, a relatively fast (average time to response onset ~4.5 s, Figure 3A, bar chart in inset) outward current was observed as a function of time (Figure 3A). Evidence that this UV-induced current reflects the activation of the expressed TRPL channels came from measurements of a family of i-V curves from cells that were kept in the dark only ~6 min before UV illumination. HEK cells in which we waited only a few min (<6 min) in the dark before the application of the UV light revealed i-V curves with amplitudes that increased with time (Figure 3B). We hypothesize that the increasing amplitudes of the i-V curves with time (Figure 3B) reflect the accumulation of active OptoDArG in the surface membrane of the recorded cell. The observed outward rectification and ~0 mV reversal potential of the i-V curves after UV illumination supports the identification of this current as a TRPL-dependent current. The application of the intense UV light to un-transfected HEK cells or to TRPL expressing cells with OptoDArG in the recording pipette, which were illuminated after a short (<3 min) dark interval after whole cell formation, did not elicit the above current (Figure 3C). These experiments indicated that similar to the inclusion of OAG in the recording pipette (Figure 2B,D), the inclusion of inactive OptoDArG in the recording pipette requires >10 min to accumulate in the surface membrane of the cells to allow the induction of large currents by UV light. Strikingly, photo-activation of the accumulated OptoDArG in the surface membrane dramatically accelerated the kinetics of TRPL-dependent current formation ~100-fold relative to OAG (Figure 2D). Nevertheless, the ~5 s delay in TRPL current activation by OptoDArG (Figure 3A) is still more than two orders of magnitude slower than the latency of TRPL activation in *Drosophila* photoreceptors by light (Figure 3D).

### 2.4. A Mutation in the TRPL Pore-Region Causing Constitutive Channel Activity Enhanced and Accelerated the OAG-Induced Activation of TRPL

**Figure 4:**
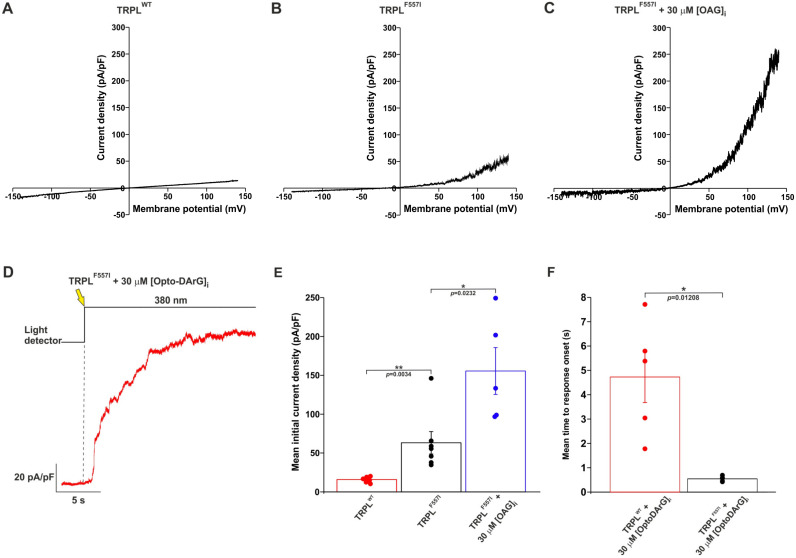
Intracellular OAG enhanced and accelerated the initial basal current of the constitutively active TRPL^F557I^ mutant channel. (**A**) A representative control i-V curve of the initial current obtained from patch clamp whole cell measurements from a T-REx-293 cell expressing TRPL^WT^ and GFP, in response to voltage ramps from −150 mV to +150 mV. (**B**) Representative i-V curve of the initial current obtained from patch clamp whole cell measurements from a T-REx-293 cell expressing TRPL^F557I^ mutant channel and GFP, in response to voltage ramps from −150 mV to +150 mV. (**C**) Representative i-V curve of the initial current obtained from patch clamp whole cell measurements from a T-REx-293 cell expressing TRPL^F557I^ and GFP, in response to voltage ramps from −150 mV to +150 mV applied every 5 s. The i-V curves were measured 18 ± 1.2 s after whole cell formation while the pipette solution included 30 μM OAG. (**D**) A representative current as a function of time showing an outward current induced by an intense UV (380 nm) light applied to an HEK cell expressing the TRPL^F557I^ mutant channel, in which 30 μM OptoDArG was included in the intracellular solution of the patch clamp pipette. The UV light was applied ~6 min after whole cell formation, while the cell was kept in the dark during that time to keep the OptoDArG in its inactive *trans* configuration. Then, the membrane voltage was increased in the dark from 0 mV to +100 mV holding potential and the UV light was turned on, resulting in a robust outward current (red trace). The upper black trace depicts the onset of the UV (380 nm) light measured by a light detector. The yellow arrow and the dashed line indicate the time of UV light onset. (**E**) A bar chart showing the mean initial current density measured at +140 mV from T-REx-293 cells expressing TRPL^WT^ and GFP (red, *n* = 6) and cells expressing mutant TRPL^F557I^ and GFP with (blue, *n* = 5) and without the intracellular application of 30 μM OAG (black, *n* = 7). Error bars show SEM. *p* values were calculated using a two-tailed Mann–Whitney *U*-test and are indicated below the bars. Values from individual experiments are shown for each of the columns (circles). (**F**) A bar chart comparing the mean time to response onset from the time of UV light switched on in HEK cells expressing the TRPL^WT^ channel (red, *n* = 5) and the TRPL^F557I^ mutant channel (black, *n* = 5), when 30 μM of OptoDArG was included in the intracellular solution of the patch pipette. Error bars show SEM. *p* values were calculated using a two-tailed Mann–Whitney *U*-test and are indicated below the bars. Values from individual experiments are shown for each of the columns (circles).

A random chemically induced mutations screen in *Drosophila* caused several mutations in the TRP open reading frame, leading to fast retinal degeneration [1]. In later studies, it was found that the one critical missense mutation causing retinal degeneration was the F550I mutation. Interestingly, in the retinal degeneration mutant designated *trp^P^*^365^, the F550I pore-region mutation also gave rise to robust constitutively active channels [30,31]. This mutation induced cell death, most likely due to constant calcium influx through the open TRP channel [32,33]. In the present study, we exploited the F550 site of TRP that is conserved in TRPL [34]. Indeed, the homologue F557I mutation in the pore-region of TRPL also caused constitutive (but mild) activity of the mutant TRPL channel expressed in HEK cells when compared to the wild type (WT) channel (Figure 4A,B,E). Thus, the constitutive TRPL^F557I^-dependent current is rather small when compared to the equivalent current observed in the TRP^F550I^ of the *trp^P^*^365^ *Drosophila* mutant [31], but it is still significant relative to the control (Figure 4E, black vs. red). Since the F557 site residing in the pore region of TRPL [34] and DAG was suggested to operate in the pore region of the TRPC3 channel [27], we attempted to search for a possible interaction between OAG and the F557I mutation of TRPL. To this end, we applied OAG into the whole-cell recording pipette and recorded the TRPL^F557I^ dependent current. Strikingly, a large increase in the constitutive TRPL^F557I^ dependent current was observed in these cells when 30 μM OAG was introduced into the intracellular solution (Figure 4C,E) shortly (<18 ± 1.2 s) after whole cell formation. Interestingly, the enhanced amplitude of OAG-induced i-V curves of the TRPL^F557I^ mutant declined with time to low levels due to an unknown reason. Technically, 18 ± 1.2 s represents the fastest average time point that we were able to achieve from whole cell formation to the recording onset. Therefore, we thought of using OptoDArG to examine the exact time for TRPL^F557I^ channel activation by the DAG analogue. When we performed the same activation protocol for OptoDArG detailed in Section 2.3 on cells expressing the TRPL^F557I^ mutant channel, we observed a significant reduction in the time from the UV light application to the time of the response onset (mean time to response onset, Figure 4D) when compared to cells expressing the wild type TRPL channel (Figure 4F). Importantly, this mean time to response onset (542 ± 49 ms) of the TRPL^F557I^ is similar to that observed for the WT TRPC3 channel [27] (see Section 3).

## 3. Discussion

Physiological activation by light of the founding members of the TRP channels superfamily, the *Drosophila* TRP and TRPL channels, requires the activation of PLC [1]. The hydrolysis of PIP_2_ by PLC is a crucial step in the still-unclear light activation of the TRP/TRPL channels. Therefore, the generation of DAG by PLC and its putative accumulation following DAG kinase inhibition by the *rdgA* mutation have suggested the involvement of DAG in the light excitation of the channels [10]. The major light activated *Drosophila* channel, the TRP channel, cannot be functionally expressed in tissue culture cells. In contrast, the *Drosophila* TRPL channel can be readily expressed in tissue culture cells, allowing a detailed study aiming to explore the possible activation of the expressed TRPL channel by DAG. Interestingly, the exogenous application of the DAG analogues OAG or SAG to TRPL channels expressed in HEK cells did not activate the channels, while the bath application of DAG analogues did activate the mammalian TRPC3 and served as a positive control ([20] and Figure S2). This result, together with the present experiments (Figure S2), suggests that although both TRPL and TRPC3 belong to the same TRPC subfamily, there is a profound difference between TRPL and TRPC3 in the ability of DAG analogues to activate the channels via bath application. Understanding this difference may require solving the atomic structure of the *Drosophila* TRPL channel.

In the present study, we re-examined the ability of DAG analogues to activate the expressed TRPL channels in HEK cells. Unlike previous attempts to activate both the isolated *Drosophila* ommatidia and expressed TRPL channels by DAG application to the bathing solution, in this study we applied the DAG analogue to the cytosol via the patch clamp pipette. Strikingly, the intracellular application of the DAG analogue OAG robustly activated the expressed TRPL channels. By performing careful controls, we ensured that the heterologously expressed TRPL channels reached the surface membrane, and the channels could be activated by an endogenous G-protein and PLC cascade using CCh activation of the expressed muscarinic receptor. Moreover, CCh activation generated the typical TRPL-dependent current with the typical reversal potential and outward rectification (Figure 1). In control experiments, we ensured that the observed outwardly rectifying current did not arise from the activation of endogenous HEK cell channels under our experimental conditions (Figure S1). However, when induced by CCh or OAG, the initial TRPL channel activation time and the time from response onset to maximal response were much slower than the physiologically activated TRPL current induced by light in *Drosophila* photoreceptors (Figure 3D). We hypothesize that the similarity in the time to response onset between CCh and OAG activation reflects the accumulation time of a sufficient concentration of DAG necessary to activate the TRPL channels, while the time from response onset to peak amplitude reflects the continued accumulation of DAG in the vicinity of the expressed TRPL channels. In the native *Drosophila* photoreceptors, DAG is produced in the tiny, limited space of the signaling compartment membrane, generating a high DAG concentration in a millisecond timescale, resulting in rapid response kinetics. Since the accumulation of exogenously applied OAG in the plasma membrane of both *Drosophila* excised rhabdomeral membrane and the surface membrane of HEK cells is inherently slow, we overcame this difficulty by using an optically activated DAG analogue. The recent development of light-controlled lipid structures such as OptoDArG, which is based on a fast (picoseconds) trans-to-cis conversion of azobenzene [26], has enabled researchers to temporally control the active quantity of lipid mediators in membranes [27]. To use OptoDArG in our experiments, we included the inactive *trans* OptoDArG in the intracellular solution of the recording patch clamp pipette, and waited >10 min in the dark to allow the diffusion of the inactive OptoDArG and its accumulation in the surface membrane of the recorded HEK cell. A fast application of intense UV light to the recorded cell resulted in a robust and relatively fast typical TRPL-dependent current. The inability to generate a similar UV-induced current when inactive OptoDArG was included in the pipette when the UV light was applied <3 min after formation of the whole cell recordings suggests that the inactive OptoDArG applied via the pipette solution needs more than several min to reach a sufficient amount capable of inducing the TRPL dependent current upon UV illumination.

The enhanced and accelerated activation of the TRPL channels by OAG applied to the constitutively active TRPL mutant channel, TRPL^F557I^, suggests that a lower concentration of OAG is probably required to enhance the TRPL-dependent current when the channels are already in an active state. It further suggests that OAG operates in the pore region of the TRPL channel, because the F557I mutation resides in transmembrane helix 5 (S5) at the pore region. The TRPL channel belongs to the TRPC subfamily, in which the atomic structure of TRPC3 was solved by cryo-EM [35] and OAG is known to bind to the pore region ([27,36]). The results thus suggested that OAG inclusion in the recording pipette exerted its effect on the TRPL channel via the putative pore region of TRPL, supporting the notion that DAG is involved in TRPL channel activation.

The mechanism by which DAG activates TRPC3 channels is not fully understood [36]. However, studies suggest that DAG activates TRPC3 channels by directly binding to a specific site on the channel protein [37]. In the work where Groschner and colleagues first introduced the OptoDArG [27], they showed that, on average, it took ~450 ms to activate the TRPC3 channel from the time of UV application. Hence, it seems that although the *trans*-to*-cis* photo-conversion of azobenzene is in the picosecond timescale [26], the activation of the TRPC3 channel takes place within ~450 ms. In our experiments, we observed wild-type TRPL activation after ~4.5 s on average after the UV light was switched on, which is much slower than the time required for the *trans*-to*-cis* photo-conversion of OptoDArG and for TRPC3 channel activation. However, when applying the OptoDArG activation protocol to the TRPL^F557I^ mutant channel, we achieved an activation time similar to that observed in the TRPC3 channel, suggesting that 450–500 ms is the time required for the DAG-activation of TRPC channels when acting in isolation.

Our data showed that DAG can activate the expressed TRPL channel, and thus it may constitute a putative second messenger of excitation of the light-sensitive TRPL channels (see Figure S4). This conclusion supports the DAG activation of the native TRP/TRPL channels obtained in excised, inside-out patch clamp recordings from *Drosophila* photoreceptor cells [23,24]. Nevertheless, the DAG activation in both cases was much slower (~3 orders of magnitude) than the physiological activation of the TRPL/TRP channels by light. We hypothesize that the time to response onset (Figure 2D) reflects the accumulation time of a sufficient concentration of exogenously applied DAG necessary to activate the TRPL channel, while the time from response onset to peak amplitude (Figure 2E) reflects the continued accumulation of DAG in the vicinity of the channels. Thus, the apparent high concentration of DAG in the channel vicinity that is necessary for TRPL activation (Figure 2) may suggest that the sensitivity of TRPL to DAG is relatively low. We therefore hypothesize that there are at least two main mechanisms for overcoming the deduced low sensitivity to DAG: (1) a fast generation of high DAG concentrations, and (2) priming the TRPL channel for opening. There are several possible mechanisms for the priming for opening, including (i) a fast reduction in pH [38], and (ii) PIP_2_ depletion together with force-generation induced by a change in membrane lipid packing [39]. All the above mechanisms are known to accompany PLC activation. These well-described mechanisms may increase TRPL sensitivity to DAG. The high-speed light activation of TRPL in the native *Drosophila* system suggests that all of these mechanisms may operate under physiological conditions. The results obtained from the TRPL^F557I^ mutant are consistent with the above hypothesis, since this mutant channel is presumably “primed for opening” as reflected by a small but significant increase in its initial basal current (Figure 4). Thus, our ability to further reduce the TRPL activation time by OptoDArG when using the F557I mutant channel (Figure 4), and the similarity of this activation time to the TRPC3 activation time by OptoDArG, also strengthen our hypothesis regarding TRPL activation by DAG.

To conclude, in this study we succeeded in demonstrating the direct DAG activation of the TRPL channel. The kinetics of the DAG-induced TRPL-dependent current were slow, most likely because of the inherent slow accumulation of a large concentration of a DAG analogue in the surface membrane. Therefore, we also used the DAG analogue OptoDArG that adopts the inactive *trans* conformation in the dark and was able to slowly accumulate in the surface membrane, and which was then rapidly converted into the active *cis* conformation by UV light. However, the relatively fast (~5 s) induction of the TRPL-dependent current by OptoDArG and its acceleration by a pore mutation of TRPL was still considerably slower than the physiological light-induced TRPL current, suggesting that DAG alone is not sufficient for TRPL channel activation under physiological conditions.

## 4. Materials and Methods

### 4.1. Fly Stocks

*Drosophila melanogaster* flies were raised at 24 °C in a 12 h dark/light cycle. Both male and female flies were used for experiments. Three strains were used in this study: W^118^; (referred to as WT), *yw*;; *trp^P^*^343^ (referred to as *trp^P^*^343^, [40]) and *yw*; *trpl*^302^, cn, bw; (referred to as *trpl*^302^, [41]).

### 4.2. Cell Culture

T-REx^TM^-293 cells (Thermo-Scientific, Waltham, MA, USA) were grown at 37 °C, 5% CO_2_ in Dulbecco’s modified Eagle’s medium (DMEM, 01-055-1A, Biological Industries, Beit-Haemek, Israel) supplemented with 10% TET-System approved fetal bovine serum (04-005-1A, Biological Industries), 1% Penicillin-Streptomycin (03-031-1B, Biological Industries), 2 mM L-glutamine (03-020-1B, Biological Industries) and 5 mg/mL Blasticidin (ant-bl-1, InvivoGen, San Diego, CA, USA). Cells were not used above passage 30. Transfections were performed using TransIT-LT1 (MC-MIR-2300, Mirus Bio, Madison, WI, USA) transfection reagents, according to the manufacturer’s protocol. Tetracycline was added 18–24 h prior to experiments to achieve maximum saturating levels of plasmid expression.

### 4.3. Plasmids Used in This Study: See Table 1

The following table includes a list of all the plasmids used in this study and a short description of what they were used for.

### 4.4. Antibodies

The primary antibodies used in this study were: rabbit polyclonal anti-TRPL (from Dr. A. Huber) and mouse monoclonal anti-beta actin (ab8224, Lot:GR221876-7, Abcam, Waltham, MA, USA). The secondary antibodies used in this study were: peroxidase affinipure goat anti-mouse (115-035-003, Lot:139283) and goat anti-rabbit (111-035-003, Lots:115298, 139196) IgG (H+L). All secondary antibodies were purchased from Jackson ImmunoResearch, Cambridgeshire, UK.

### 4.5. Western Blot Analyses

For protein isolation from T-REx-293 cells*:* pcDNA4-TRPL^WT^-T-REx-293 and naive T-REx-293 cells were washed twice with 5 mL of ice-cold PBS and detached using 5 mL of ice-cold PBS (without Ca^2+^ or Mg^2+^) and a scraper. Cells were centrifuged at 300× *g* for 5 min at 4 °C, and supernatant was removed. The cell pellet was solubilized on ice using lysis buffer (1% Triton X-100, 25 mM Tris–HCl (pH = 7.5), 150 mM NaCl and 5 mM EDTA together with a protease inhibitor cocktail (1:100, P8340 Sigma) and overhead for 1 h at 4 °C. Lysates were centrifuged at 12,000× *g* for 20 min at 4 °C and the supernatant was collected.

For protein isolation from Drosophila eyes: 15 dark raised, newly eclosed heads of WT, *trpl*^302^ and *trpl^P^*^343^ flies were homogenized in lysis buffer with protease inhibitor cocktail. Head homogenates were extracted on ice for 30 min then centrifuged at 12,000× *g* for 20 min at 4 °C, and the supernatant was collected.

Laemmli buffer was added to all the supernatants, and samples were boiled at 95 °C for 5 min and separated using 8% SDS-PAGE gel. Proteins were transferred for 1 h at 350 mA to PVDF membranes in Tris-glycine buffer supplemented with 20% methanol. The membranes were then blocked with 4.5% BSA (A7030). The blots were probed with anti-TRPL and anti-beta actin primary antibodies that were then bound to secondary antibodies conjugated to peroxidase. Signals were detected using EZ-ECL reagents (Biological Industries).

### 4.6. Confocal Imaging

Images of cells were acquired using a confocal microscope (Zeiss, Oberkochen, Germany, LSM 980 with Airyscan 2) with a Zeiss Plan-Apochromat 63×/1.4 oil DIC M27 objective controlled by the ZEN software (version 3.3 blue). Cells were seeded on 35 mm plates (FD-35-100, WPI) coated with Poly-lysine. Confocal images were taken from T-REx-293 cells co-transfected with pCDNA4-hTRPC3-mCherry and pCDNA4-dTRPL-GFP. Laser irradiation at 488 nm was used to excite the GFP and imaged using a 490–550 nm band pass emission filter. Detector gain and laser intensity were constant. Super resolution images were also taken from the cells using the Airyscan mode to achieve resolution up to 140 nm. Laser irradiation at 488 nm and 561 nm was used to excite the GFP and mCherry, respectively. GFP was imaged using a 420–480 nm and a 495–550 nm band pass emission filter and mCherry was imaged using a 574–720 nm band pass emission filter.

### 4.7. Electrophysiology

Cells were seeded on Poly-Lysine coated 16 mm coverslips. Whole-cell currents were recorded at RT using borosilicate patch pipettes of 3–5 MΩ resistance. The time from whole cell formation was recorded continuously, in order to determine the time to response onset and the time from onset to maximal current. Electrical signals were amplified using Axopatch 1D (Axon Instruments, San Jose, CA, USA) and data were captured using a Digidata 1440A (Molecular Devices, San Jose, CA, USA) interfaced to a computer. Membrane potential was held at 0 mV and currents were measured in response to 1 s voltage ramps from −150 to +150 mV every 5 s, or membrane potential was held at +100 mV and currents were measured in a gap-free protocol. UV 380 nm light was given using a Xenon lamp with an ET380× Chroma filter, and was manually controlled through a Lambda 10-B shutter (Sutter Instruments, Novato, CA, USA).

### 4.8. Solutions

Intracellular solution: see Table 2.

Extracellular solutions: see Table 3 and Table 4.

CsMeSO_3_ (C1426), CsCl (289329), CsOH (C8518), NaCl (7548-4400, DAEJUNG, Busan, Korea), MgCl_2_ (M9272), Na-gluconate (G9005), KCl (P5405), CaCl_2_ (C7902), EGTA (E4378), MgATP (A9187), NaGTP (G8877), HEPES (H3375), NaOH (7708-10, Mallinckrodt Chemicals, Dublin, Ireland), HCl (H1758), D-glucose (1916-01, J.T.Baker, Phillipsburg, NJ, USA). All chemicals were purchased from Sigma Aldrich unless stated otherwise.

### 4.9. Pharmacology

Carbachol (PHR1511, Sigma, St. Louis, MO, USA), GaCl (G7532, Sigma), 1-Oleoyl-2-acetyl-sn-glycerol (495414, Sigma); OptoDArG (provided by Dr. K. Groschner).

### 4.10. Statistics

Statistical analyses were conducted using Prism 8. Statistical significance was determined using a two-tailed Mann–Whitney U-test (* *p* < 0.05, ** *p* < 0.01, *** *p* < 0.001, ns—not significant *p* > 0.05).

All experiments were conducted in E1 SES, unless otherwise stated.

## Figures and Tables

**Table 1:** Plasmids used in this study.

Plasmid Name	Used for
pcDNA4-TRPL^WT^-GFP	Protein expression in confocal imaging
pcDNA4-hTRPC3-mCherry	Protein expression in confocal imaging and electrophysiological experiments
pcDNA4-TRPL^WT^	Protein expression in electrophysiological experiments and Western blot analysis
pcDNA4-GFP	Protein expression in electrophysiological experiments
pcDNA4-hM1R	Protein expression in electrophysiological experiments
pcDNA4-TRPL^F557I^	Protein expression in electrophysiological experiments

**Table 2:** I1 Intracellular solution (CsMeSO_3_).

Chemical Name	Concentration (mM)
CsMeSO_3_	120
CsCl	25
MgCl_2_	1
EGTA	5
MgATP	3
NaGTP	0.3
HEPES	10

pH 7.2, adjusted with CsOH.

**Table 3:** E1 Standard Extracellular Solution (SES).

Chemical Name	Concentration (mM)
NaCl	147
KCl	5
MgCl_2_	1
HEPES	10
D-glucose	10

pH 7.4, adjusted with NaOH.

**Table 4:** E2 Extracellular Solution (Na-Glu) [42].

Chemical Name	Concentration (mM)
NaCl	10
MgCl_2_	1
HEPES	5
EGTA	1.5
Na-gluconate	140

pH 7.3, adjusted with NaOH.

## Data Availability

The data presented in this study are available on request from the corresponding author. The data are not publicly available due to privacy limitations.

## Supplementary Materials

The supporting information can be downloaded at: https://www.mdpi.com/article/10.3390/ijms24076289/s1. (see Refs. [20,22,43,44,45,46,47]).
