# Supplementary material for: Diacylglycerol Activates the Drosophila Light Sensitive Channel TRPL Expressed in HEK Cells
Source: Int J Mol Sci. 2023 Mar 27;24(7):6289. doi: 10.3390/ijms24076289 (PMC10093889; doi:10.3390/ijms24076289)
Supplement: Supplementary file 1 [file ijms-24-06289-s001.zip › ijms-2256287-supplementary.pdf]

Supplement

It has been reported that HEK cells endogenously express muscarinic (M3) receptor and DAG-activated TRPC3 channels, which can be activated by CCh via the endogenous cascade that includes M3, Gq and PLCβ [43]. We, therefore, examined whether an endogenous Gq, PLCβ, TRPC3 cascade is significantly activated in our HEK cell preparation. To this end we applied CCh to HEK cells expressing TRPL but not hM1R (**Figure S1C,D**). We also applied CCh to HEK cells expressing hM1R but not TRPL (**Figure S1E,F**). We found that only negligible currents were observed during ~ 20 min of CCh application to the above cells (**Figure S1G)**. To verify that TRPL channels were functionally expressed in **Figure S1C,D** (but not in **Figure S1E,F**), we applied linoleic acid (a PUFA, known to potently activates TRP and TRPL channels [44], [20]) to the bath at the end of the experiments and observed the typical TRPL dependent current (data not shown). These results indicate that the CCh-induced current observed in HEK cells co-expressing the TRPL channels together with hM1R (**Figure S1A,B,G)** did not arise from activation of endogenous mammalian TRPC channels, but rather from M1R activation of TRPL via the endogenous Gq, PLCβ cascade.

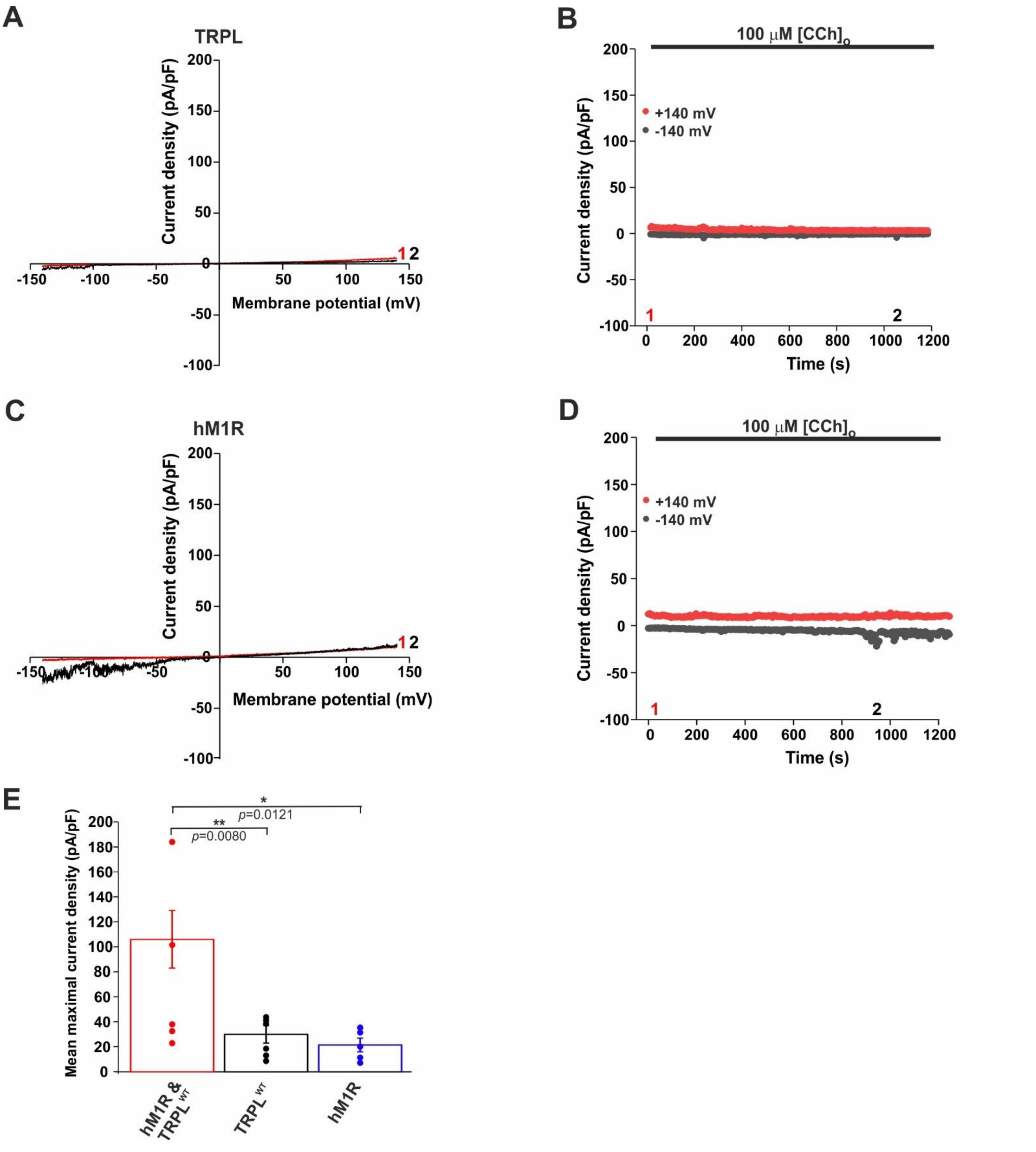

Figure S1. CCh does not elicit any significant currents when applied to cells expressing only the TRPL channel or only the M1 receptor.

- **A.** Representative i–V curve obtained from patch clamp whole cell current measurements from a T-REx-293 cell expressing dTRPL^{WT} and GFP (but no hM1R) after bath application of SES (red, 1) and of 100 μM CCh (black, 2).
- **B.** Corresponding currents at +140 mV and -140 mV during 20 min of recording. Numbers indicate the time of the selected i-V curves.
- **C.** Representative i–V curve obtained from patch clamp whole cell current measurements from a T-REx-293 cell expressing hM1R and GFP (but no dTRPL^{WT}) before bath application of CCh (red, 1) and after 100 μM CCh (black, 2).
- **D.** Corresponding currents at +140 mV and -140 mV during 20 min of recording. Numbers indicate the time of the selected i-V curves.

E. A bar chart showing the mean maximal current density measured after bath application of 100 μM CCh in T-REx-293 cells expressing $dTRPL^{WT}$, GFP and hM1R (red, n=6), $dTRPL^{WT}$ and GFP (black, n=6), and hM1R and GFP (blue, n=5). Error bars show SEM. *P* values were calculated using a two-tailed Mann-Whitney *U*-test and are indicated below the bars. Values from individual experiments are shown for each of the columns (circles).

It has been well established that exogenous bath application of a DAG analogue to tissue culture cells expressing mammalian TRPC3 or TRPC6 activates the expressed channels [45]. In contrast, bath application of a DAG analogue (SAG) to HEK cells expressing TRPL elicited only negligible currents, while SAG application to TRPC3 expressing HEK cells induced a robust current, serving as a positive control [20]. To reexamine our attempts to activate expressed TRPL channels, by application of a DAG analogue to the bathing solution, we applied 100 μM OAG to the bathing solution during ~30 min of whole cell recordings from HEK cells expressing TRPL channels (**Figure S2A, C**). Only small negligible currents were observed under these conditions (**Figure S2A, C**). The functional expression of TRPL channels in each of the above tested cells was verified by application of linoleic acid, the potent activator of TRPL channels [22,44] that readily induced TRPL-dependent current (data not shown, [20]). As a positive control, we examined whether OAG application to the bath is effective in activation of mammalian TRPC channels, we expressed hTRPC3-mCherry in HEK cells (see **Figure 1B**) and applied 100 μM OAG to the bath. A large TRPC-dependent current was observed **(Figure S2B, C)** as previously shown [45]. These experiments showed that there is a clear difference in DAG activation between mammalian TRPC and *Drosophila* TRPL channels.

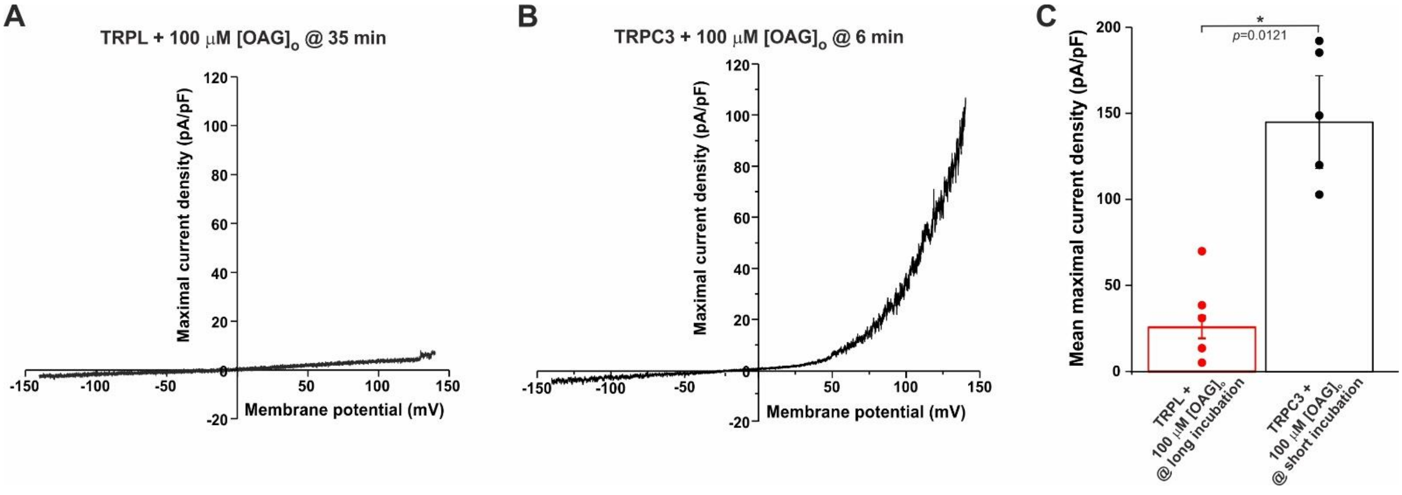

Figure S2. Bath application of OAG to cells expressing TRPL and TRPC3.

A. Representative maximal i–V curve obtained from patch clamp whole cell current measurements from a T-REx-293 cell expressing $dTRPL^{WT}$ and GFP, in response to voltage ramps from -150 mV to +150 mV, following 35 min of incubation in 100 μM OAG in E2 solution (see Materials and Methods).

B. Representative maximal i–V curve obtained from patch clamp whole cell current measurements from a T-REx-293 cell expressing hTRPC3-mCherry, in response to voltage ramps from -150 mV to +150 mV, following 6 min of incubation in 100 μM OAG in E2 solution (see Materials and Methods).

C. A bar chart showing the mean maximal current density measured from T-REx-293 cells expressing $dTRPL^{WT}$ and GFP (red, n=5) or hTRPC3-mCherry (black, n=5), after relatively long incubation (18-36 min for TRPL) or shorter incubation (6-25 min for TRPC3) in 100 μM OAG. Error bars show SEM. *P* value was calculated using a two-tailed Mann-Whitney *U*-test and is indicated below the bar. Values from individual experiments are shown for each of the columns (circles).

To ensure that the currents observed during 20 min of whole cell recordings when OAG was included in the intracellular solution were not a result of an increase of spontaneous TRPL channels activity, we performed the following control experiments: Whole cell recordings from HEK cells expressing TRPL channels *without* including OAG in the intracellular solution was performed during 20 min. Only small negligible currents were observed under these conditions (**Figure S3A, B**). The functional expression of TRPL channels in the control experiments (**Figure S3**) was verified by application of linoleic acid, the potent activator of TRPL channels [22,44] to the bathing solution of TRPL expressing cells that induced the typical i-V curve of the TRPL dependent current (data not shown, [20]). As an

additional negative control, which ensured that the observed OAG-induced currents (**Figure 2**) were not a result of putative endogenous TRPC3 channels activation, we used un-transfected HEK cells (T-Rex) in which 30μM OAG was included in the intracellular solution during 20 min. Only small negligible currents were observed under these conditions (**Figure S3C, D**).

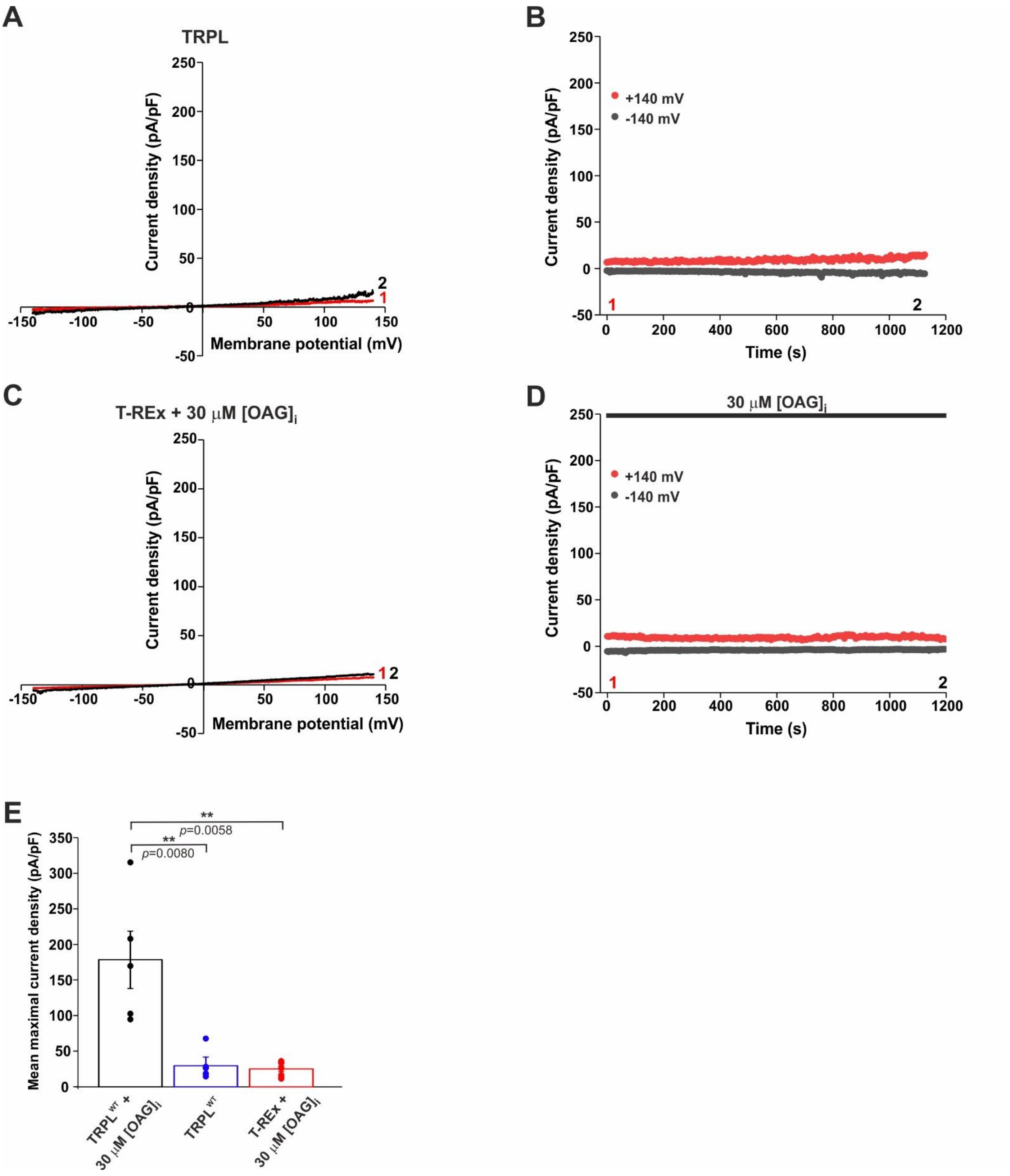

Figure S3. TRPL was not spontaneously activated during 20 min of recording, and intracellular application of OAG did not elicit any currents in naïve HEK cells.

A. Representative i–V curves obtained from patch clamp whole cell current measurements from a T-REx-293 cell expressing dTRPL^{WT} and GFP, in response to voltage ramps from -150 mV to +150 mV, during 20 min of recording, at different time points.

B. Corresponding currents at +140 mV and -140 mV. Numbers indicate the time of the selected i-V curves.

C. Representative i–V curves obtained from patch clamp whole cell current measurements of an un-transfected T-REx-293 cell, in response to voltage ramps from -150 mV to +150 mV, during 20 min of intracellular application of 30 μM OAG at different time points.

D. Corresponding currents at +140 mV and -140 mV. Numbers indicate the time of the selected i-V curves. Numbers indicate the time of the selected i-V curves.

E. A bar chart showing the mean maximal current density measured after intracellular application of 30 μM OAG via the patch clamp pipette in cells expressing dTRPL^{WT} and GFP (black, n=5, see Figure 2C). The bar chart also shows mean maximal current density measured in un-transfected T-REx cells after intracellular application of 30 μM OAG via the patch clamp pipette during ~20 min of recording (red, n=7), or after 20 min of recording from cells expressing dTRPL^{WT} and GFP (blue, n=6). Error bars show SEM. *P* values were calculated using a two-tailed Mann-Whitney *U*-test and are indicated below the bars. Values from individual experiments are shown for each of the columns (circles).

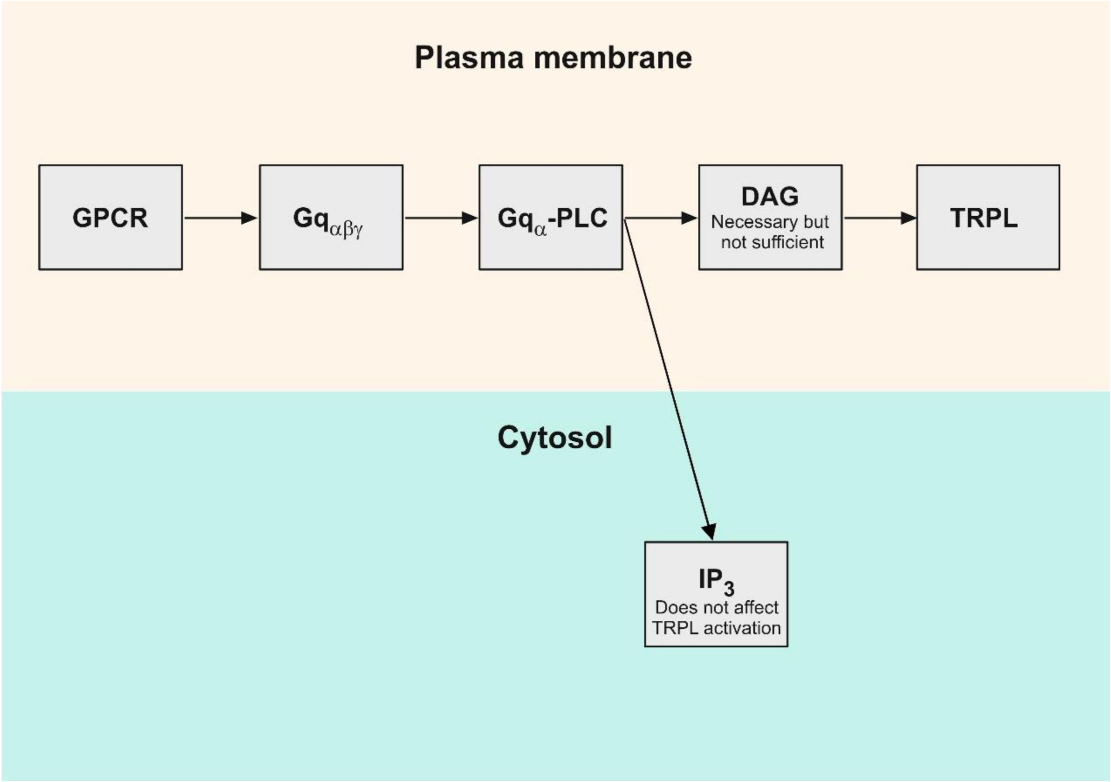

Figure S4. A schematic representation of the proposed DAG activation cascade of TRPL expressed in HEK cells.

The G-protein coupled receptor (GPCR) is activated by an agonist and leads to the activation of the native HEK heterotrimeric G-protein ($Gq_{\alpha\beta\gamma}$) by promoting the GDP to GTP exchange on the Gqα. In turn, this causes the activation of phospholipase Cβ (PLC), which hydrolyzes PIP_2 into the soluble IP_3 and the membrane-bound DAG. The IP_3 has no role in TRPL channel activation [46,47]. DAG remains in the plasma membrane and promotes TRPL channel opening.
